# Consistent population declines but idiosyncratic range shifts in Alpine orchids under global change

**DOI:** 10.1038/s41467-020-19680-2

**Published:** 2020-11-17

**Authors:** Costanza Geppert, Giorgio Perazza, Robert J. Wilson, Alessio Bertolli, Filippo Prosser, Giuseppe Melchiori, Lorenzo Marini

**Affiliations:** 1 DAFNAE, University of Padova, Viale dell’Università 16, 35020 Legnaro, Padova Italy; 2 Museo Civico di Rovereto, Borgo Santa Caterina, 41, 38068 Rovereto, Trento Italy; 3 Departamento de Biogeografía y Cambio Global, Museo Nacional de Ciencias Naturales (MNCN-CSIC), Madrid, 28006 Spain

**Keywords:** Biodiversity, Climate-change ecology

## Abstract

Mountains are plant biodiversity hotspots considered particularly vulnerable to multiple environmental changes. Here, we quantify population changes and range-shift dynamics along elevational gradients over the last three decades for c. two-thirds of the orchid species of the European Alps. Local extinctions were more likely for small populations, after habitat alteration, and predominated at the rear edge of species’ ranges. Except for the most thermophilic species and wetland specialists, population density decreased over time. Declines were more pronounced for rear-edge populations, possibly due to multiple pressures such as climate warming, habitat alteration, and mismatched ecological interactions. Besides these demographic trends, different species exhibited idiosyncratic range shifts with more than 50% of the species lagging behind climate warming. Our study highlights the importance of long-term monitoring of populations and range distributions at fine spatial resolution to be able to fully understand the consequences of global change for orchids.

## Abstract (web summary)

Many mountain species are threatened by climate change and habitat loss. Here, the authors investigate population declines and range shifts of orchids in an alpine region in NE Italy over 28 years. For most species, population size decreased, while range shifts were idiosyncratic with over half of the species lagging behind climate change.

## Introduction

Mountain ecosystems harbour a high rate of endemic and rare plant species that are considered particularly vulnerable to climate change^1^. While a large body of research has elucidated how plants respond to temperature warming by shifting their range^2–4^, significant knowledge gaps still remain. First, there is growing evidence that range shifts can lag behind climate change for several decades due to the ability of plants to persist under unfavourable conditions, dispersal limitation or lack of suitable habitats^5–7^. Declines in population density stemming from changes in mortality and fecundity rates are expected to precede range shifts^8,9^, and even when demographic changes are dramatic, they may often go undetected due to the lack of long-term monitoring data^10,11^. Second, current methodological approaches such as resurveys of permanent sites or species distribution modelling are often limited to common and abundant taxa, overlooking the response of rare species^7^. Third, previous research has mostly focussed on population dynamics at mountain tops, where warm-adapted species are expanding their distributions but cold-adapted species tend to decline in abundance or to go extinct due to climate warming^12^. There is also an urgent need to consider the dynamics of species at the rear, low-elevation edge of their distributions^13,14^, where the pressures of global change are likely to be stronger and the effects of climate warming are less predictable due to the co-occurrence of multiple drivers of plant distribution^15^.

Besides climate warming, mountain ecosystems have experienced rapid habitat transformations such as forest expansion, increased urbanization and invasion of exotic species^2,16^, with potentially negative consequences for resident plant communities. In the European Alps, a major trend is the abandonment of remote and less productive areas at mid-elevations and above^17^. Human activities directly shape the elevational distribution of habitats, often irrespective of the direction and speed of climate change^15,18^. As a result, climate warming may cause a spatial mismatch between suitable climatic conditions and habitat availability^19–21^. Under these circumstances, habitat distribution and quality are expected to play a central role in explaining local population dynamics and climate-induced range shifts^22–26^, in particular for specialist, rare and threatened species whose range shift dynamics are likely to be most sensitive to the elevational distribution of suitable habitats.

**Fig. 1:**
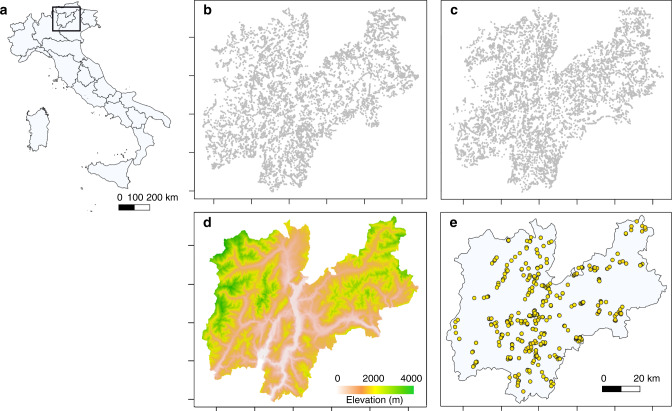
Spatial location of orchid occurrence. **a** Location of the study area, **b** geographical distribution of the sites (grey points) over the first period (1990–2003: *n* = 10,293) and **c** the second period (2004–2017: *n* = 11,308), **d** digital elevation model of the study area (resolution: 25 × 25 m) and **e** location of the 463 resurvey sites (yellow points).

Here we analysed population survival, trends in population size, and range-shift dynamics of Alpine orchids over 28 years across the whole elevational range (66–2970 m) in one of the plant diversity hot-spots of Europe (Italy, Trentino) (Fig. 1). Orchids are one of the most threatened groups of plants, and population declines are well documented worldwide^27–30^ (but see ref. ^31^ for a positive effect of warming on orchid populations). These declines are usually associated with land-use intensification or habitat loss^32,33^, coupled with the loss of mutualistic interactions with mycorrhizal fungi and pollinators^28^. Moreover, plant species in the southern European Alps are expected to shift upwards with a rate of 3.8–5.5 m year^−1^ to keep track of recent rates of warming^2^. We used multiple data sets containing a very large number of both occurrence records and population data for taxa that are normally disregarded due to their rarity and scattered geographical distribution. First, we combined historical data with a field resurvey campaign to elucidate the mechanisms underpinning orchid population persistence under global change. Second, we quantified both orchid demographic trends and shifts in the optimum, rear and leading edge of species’ elevational ranges. Here we show that orchid populations at the rear edge and in sites undergoing habitat alteration were more likely to suffer local extinctions. Similarly, population size declined at the rear edge of the elevation range in most habitat types, contributing to increased local extinction risk. Besides these consistent population declines, different species exhibited idiosyncratic range shifts with upward, downward and no movement, suggesting that temperature was not the sole factor driving range dynamics. Despite some upward shifts, the interspecific variability in range dynamics meant that most species did not shift their range uphill as fast as rates of warming.

## Results and discussion

### Orchid habitat preference

**Fig. 2:**
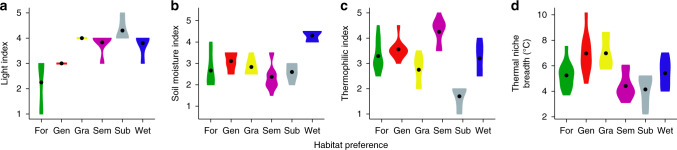
Ecological characterization of habitat preference categories. Landolt ecological indicator values^60^ for **a** light (from very shady: 1 to very bright: 5), **b** soil moisture (from very dry: 1 to flooded: 5), **c** temperature (from alpine: 1 to very warm: 5) and **d** realized breadth of the thermal niche in the study area based on annual mean temperature. Habitat preference categories: Forest (For), Generalist (Gen), Grassland (Gra), Semi-natural grassland (Sem), Subalpine (Sub), Wetland (Wet). Violin plots were drawn using the geom_violin() function with default settings in the ggplot2 package in R. Points represent medians.

**Fig. 3:**
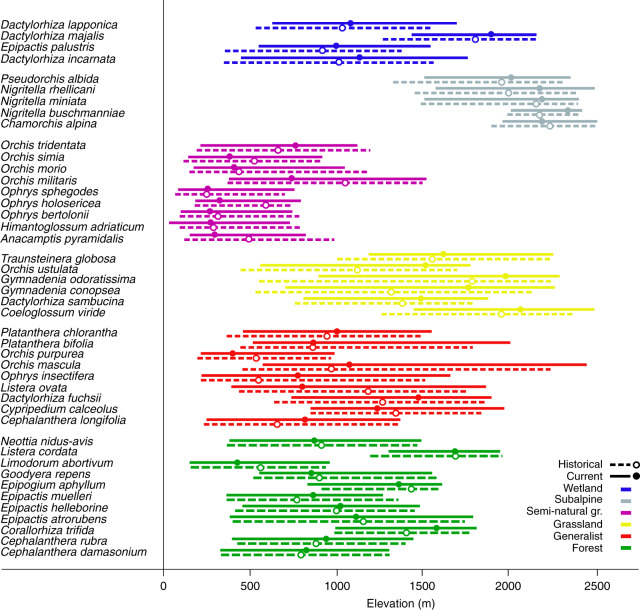
Orchid elevational distribution. Elevational distribution for the orchid species with at least 30 distribution records per period (historical, 1990–2003, and current, 2004–2017) pooled by habitat preference. Dashed bars represent historical (1990–2003) and solid bars current (2004–2017) rear (5%) and leading edges (95%), points represent optima (highest peak) of the density distribution.

As most orchid species are specialists with clear preferences for a particular habitat type^28^, we first attributed each species to one of the six non-overlapping categories using a published description of habitat preferences^34^ (see ‘Methods’ for details): (1) specialists of forest (forest), (2) generalists, (3) specialists of grassland habitats with wide thermal niche (grassland), (4) warm-adapted specialists of semi-natural grassland (semi-natural), (5) cold-adapted specialists of subalpine habitats (subalpine), and (6) specialists of wetlands (wetland) (Supplementary Table 1). These habitat preference categories drew together species with a similar ecology and elevational distribution. All orchid species were adapted to open areas and therefore to full light except for forest orchids and generalists (Fig. 2a). Wetland orchids were the only group associated with wet soil conditions (Fig. 2b). Consistent with their elevational distribution (Fig. 3), subalpine orchids were cold-adapted species, while species occurring in semi-natural grasslands were the most thermophilic across their geographic ranges (Fig. 2c). The remaining four groups preferred intermediate temperatures found at mid-elevations across the study area, with generalists and species living in grasslands being characterized by the widest breadth of thermal niches (Fig. 2c, d).

### Local survival based on resurveys

**Fig. 4:**
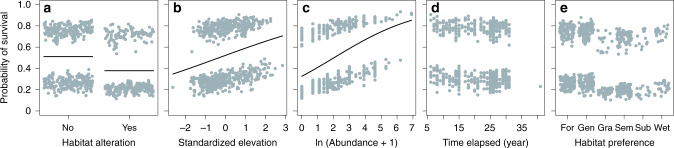
Biotic and abiotic drivers of local population extirpation. Partial residual plots showing the effect of **a** habitat alteration, **b** elevation, and **c** population size on orchid probability of occurrence in resurveyed sites. Also non-significant effects of **d** time elapsed between the initial and second survey and **e** habitat preference were reported. Elevation was standardized to mean 0 and SD 1 to make elevational ranges comparable among species, i.e. the most negative values corresponded to the rear edge and the largest positive values to the leading edge. Plots were drawn using the visreg() function with default settings in the visreg package in R.

We analysed local population dynamics of species with different habitat preferences using a resurvey approach (see ‘Methods’). In 2018 and 2019, we revisited 463 sites to test for local habitat alteration and population survival since initial surveys (average difference = 20.5 years, SD = 8.4 years). Habitat alteration was observed in 37% of the resurveyed sites and included land-use changes (e.g. abandonment of grasslands and agricultural expansion) or local disturbances related to building infrastructure. Habitat alteration tended to be more likely at lower elevations (generalized linear models (GLM) binomial, *p* = 0.063). Orchid survival was explained by habitat alteration, elevation and historical population size, irrespective of habitat preference and time elapsed between the two surveys (Table 1 and Supplementary Table 2). First, habitat alteration affected survival negatively with a probability reduction of c. 17% (Fig. 4a), supporting previous observations that land-use changes such as afforestation, urbanization and agricultural expansion are key drivers of orchid local extinction^32,35^. In particular, the observed loss and degradation of semi-natural grasslands have been related to declines in plant specialists^36^. Second, orchid populations were less likely to survive if the population was located at the lower part of the species’ elevational range (Fig. 4b). Biogeographical theory suggests that rear edge populations are at higher risk of extinction than populations at the core of the species’ range as marginal populations occupy less favourable and deteriorating climates and are also subjected to constraints, including altered biotic interactions and deterioration of genetic diversity^37^. As our model controlled for the effects of habitat alteration, the lower probability of survival at the lowest elevations suggests that climate warming could have increased the risk of extinctions at low elevations. However, other factors such as loss of biotic interactions with pollinators and mycorrhizal fungi (themselves potentially related to climate or habitat degradation) could also contribute to the observed patterns. Third, we found a positive effect of historical population size (Fig. 4c), consistent with the predicted negative relationship between population size and extinction in fragmented plant populations^38^.

**Table 1:** Effect of time (difference in years between the two surveys), historical population size, elevation, habitat alteration (yes and no) and habitat preference on the probability that the orchid population survived until the resurvey.

Fixed effects	*χ*^2^	*p*
Time	1.220	0.269
Log (size)	35.189	<0.001
Elevation	8.906	0.003
Habitat alteration	9.600	0.002
Habitat preference	8.684	0.122

We fitted generalized linear mixed models assuming a binomial distribution with species as random factor (random intercept). Elevation was standardized to mean 0 and SD 1 to make elevational ranges comparable among species.

### Temporal trends in population size

**Fig. 5:**
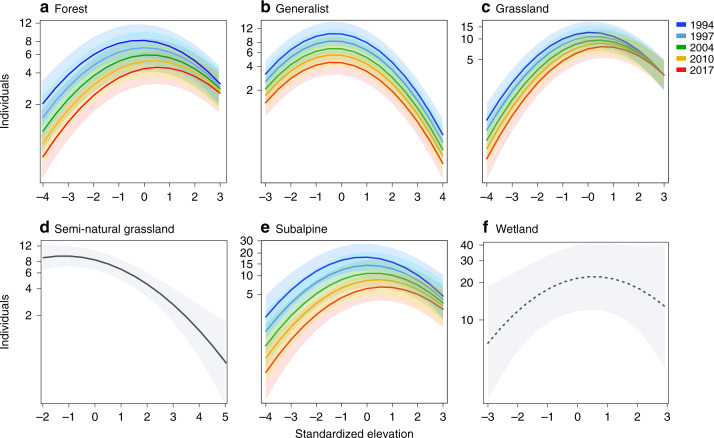
Temporal trends in orchid population size across species elevational range. Plots depicting the effect of time, elevation (linear and quadratic terms) and, if significant, their interaction on population size. Separate models were run for different habitat preference categories: **a** forest, **b** generalist, **c** grassland, **d** semi-natural grassland, **e** subalpine, and **f** wetland. Within each species, elevation was standardized to mean 0 and SD 1 to make elevational ranges comparable among species, i.e. the most negative values corresponded to the rear edge and the largest positive values to the leading edge. The relationship for species showing no effect of time is shown in black. For clarity reasons, colour coding shows only 5 years but orchid population size has been recorded over 28 years (1990–2017). Shading areas shows 95% confidence intervals around model estimates (solid line). For wetland (dashed line), the quadratic effect of elevation was marginal (*p* = 0.066, *n* = 534 observations for 4 species). Plots were drawn using the plotEffect() function in the effects package in R. Partial residuals were not shown due to the large number of data points.

By testing the effect of time (continuous), elevation and their interaction, we quantified how local population size of orchids with different habitat preferences has changed over the past 28 years across the whole elevational range. To do so, we used information from 21,601 orchid sites visited one time between 1990 and 2017. Consistent with the existence of a thermal optimum at mid-elevations, all species exhibited a hump-shaped relationship between elevation and local population size, except for wetland orchids that showed a weaker response (Table 2 and Supplementary Table 3). Population size of most species decreased in recent years, but with differences among habitat preference categories, and for some categories at different elevations (Fig. 5). In accordance with an expected negative effect of warming at the rear edge^37^, population size at the lower elevational limits of forest (Time × Elevation *p* = 0.002), grassland (Time × Elevation *p* = 0.011) and subalpine species (Time × Elevation *p* = 0.043) declined more strongly than at the upper limits, where population size showed a less pronounced decrease (Fig. 5a, c, e). Populations of generalist orchids decreased (Fig. 5b), and even if only with a marginal trend, their decline was also stronger at the rear than at the leading edge (Time × Elevation *p* = 0.066). This effect on population size is consistent with the higher probability of extinction at the rear edge observed in the resurveys (Fig. 4b). Population size did not change over time only for two groups with contrasting climate and habitat preferences. Species associated with semi-natural grasslands (Fig. 5d) and species of wetlands did not decline (Fig. 5f). The former are the most thermophilic species (Fig. 2) and presented a truncated realized thermal niche, i.e. if the lowest elevations in the study area corresponded to their temperature optimum, warmer temperatures may not have caused their decline. As most of the wetlands in the study region are located within protected areas (45% orchid populations occurring in wetlands are protected compared to an average of 25% for the other habitat types), the population size of these species may be maintained by habitat protection and favourable management. In contrast to previous studies investigating the response of common taxa^1,13,26^, no orchid group appeared to be favoured by climate warming, as even the most thermophilic species did not increase their population size in any part of their elevation range. This suggests that other drivers of population dynamics such as the loss of mutualistic interactions^39^ or habitat degradation^32^, as shown in the resurvey, may play an important role in explaining population declines.

**Table 2:** Effect of time (linear), elevation (linear and quadratic terms) and their interaction on population size for each habitat preference category, separately.

Habitat preference	Fixed effects	*χ*^2^	*p*
Forest	Time	202.756	<0.001
	Elevation	6.214	0.013
	Elevation^2^	111.223	<0.001
	Time (year) × Elevation	9.601	0.002
Generalist	Time	318.201	<0.001
	Elevation	54.868	<0.001
	Elevation^2^	284.900	<0.001
	Time × Elevation	3.390	0.066
Grassland	Time	61.820	<0.001
	Elevation	44.409	<0.001
	Elevation^2^	141.364	<0.001
	Time × Elevation	6.412	0.011
Semi-natural grassland	Time	0.509	0.476
	Elevation	33.479	<0.001
	Elevation^2^	9.794	0.002
	Time × Elevation	0.261	0.609
Subalpine	Time	109.331	<0.001
	Elevation	8.144	0.004
	Elevation^2^	46.331	<0.001
	Time × Elevation	4.090	0.043
Wetland	Time	0.031	0.861
	Elevation	2.746	0.097
	Elevation^2^	3.373	0.066
	Time × Elevation	1.329	0.249

To make elevational ranges comparable among species, elevation was standardized to mean 0 and SD 1. We fitted generalized linear mixed models assuming a Poisson distribution with species and an observation-level random factor as crossed random effects (see ‘Methods’).

### Range shifts

**Fig. 6:**
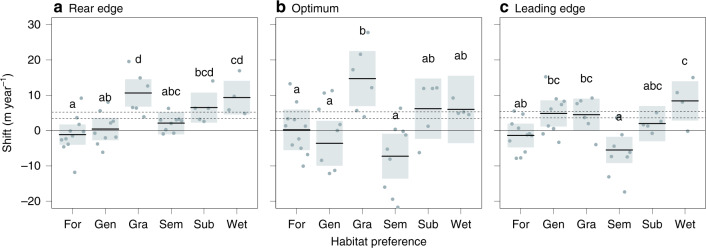
Elevation range shifts of orchids according to habitat preference. The effect of habitat preference on range shift for **a** rear edge, **b** optimum and **c** leading edge. Solid lines indicate model estimates, while shaded grey areas indicate intervals of confidence (95%). Horizontal dashed lines show the expected shift to track climate change based on the current rate of warming in the study area (3.8–5.5 m year^−1^)^2^. Forest: 11 species; generalist: 9 species; grassland: 6 species; semi-natural grassland: 9 species; subalpine: 5 species; and wetland: 4 species. Superscript letters denote significant differences (*p* < 0.05) in shift rates according to linear regression followed by Tukey’s post hoc test (see ‘Methods’). Plots were drawn using the visreg() function with default settings in the visreg package in R.

Both local extinction and demographic changes are expected to result in species range shifts^3,14^. To understand how orchid distributions changed in the past three decades, we estimated range dynamics of each species at the regional scale (see ‘Methods’). To estimate range shift, we split the historical data set into two periods (1990–2003 and 2004–2017) and evaluated species with at least 30 distribution records in each period. This approach reduced the risks that sampling biases could affect range shift estimation (see ‘Methods’). Despite some degree of inter-specific variability within habitat category, we found that orchids shifted their rear edges, optima and leading edges differently according to their habitat preference (habitat preference for rear edge *p* < 0.001, for optimum *p* = 0.001, for leading edge *p* < 0.001, Fig. 6). The rear edge shifted upwards for species inhabiting grasslands, subalpine habitats and wetlands, while species inhabiting forests, semi-natural grasslands and generalist species showed a stable rear edge (Fig. 6a). We found a similar effect of habitat preference on the optimum shift but with a larger interspecific variability (Fig. 6b). At the optimum, orchids inhabiting semi-natural grasslands exhibited a downslope movement. Finally, the leading edge shifted upwards for wetland, generalist and grassland species, while forest and subalpine orchids did not shift their leading edge (Fig. 6c). Again, semi-natural orchids shifted their leading edge downslope. Considering the average speed of temperature change in the study area (3.8–5.5 m year^−1^)^2^, rear and leading edges of forest species, optimum and leading edge of semi-natural species and optimum of generalist lagged significantly behind climate warming, while only grassland species shifted upwards faster than warming. However, only rear edges and optima of grassland orchids shifted faster than expected probably because of higher local extinctions than expected from climate warming alone.

**Fig. 7:**
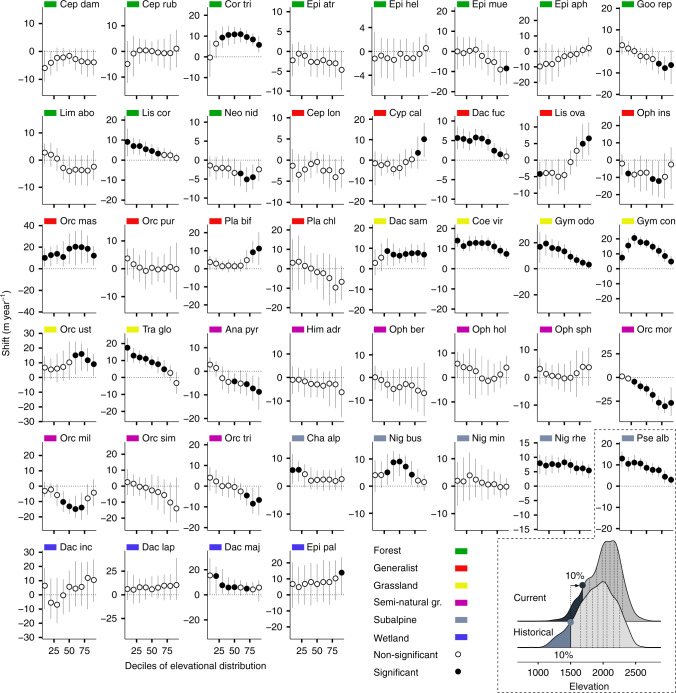
Elevation range shift between 1990–2003 (historical) and 2004–2017 (current) along the whole elevational distribution. For each species with >30 records per period, the shift for each decile between historical and current elevation density distribution is plotted (points). Vertical lines indicate 95% bootstrap confidence interval (CI) of each decile difference. Filled points indicate that the shift is different from 0 (*p* < 0.05). In the dashed outline, an example of decile comparison between the two density distributions (historical vs. current) for *Pseudorchis albida* (*Pse alb*—subalpine) is depicted: all deciles shift upwards (i.e. positive values with 95% CI not crossing the zero line) but less at the leading than at the rear edge. Abbreviations of species names: Ana pyr *Anacamptis pyramidalis*, Cep dam *Cephalanthera damasonium*, Cep lon *Cephalanthera longifolia*, Cep rub *Cephalanthera rubra*, Cha alp *Chamorchis alpina*, Coe vir *Coeloglossum viride*, Cor tri *Corallorhiza trifida*, Cyp cal *Cypripedium calceolus*, Dac fuc *Dactylorhiza fuchsii*, Dac inc *Dactylorhiza incarnata*, Dac lap *Dactylorhiza lapponica*, Dac maj *Dactylorhiza majalis*, Dac sam *Dactylorhiza sambucina*, Epi atr *Epipactis atrorubens*, Epi hel *Epipactis helleborine*, Epi mue *Epipactis muelleri*, Epi pal *Epipactis palustris*, Epi aph *Epipogium aphyllum*, Goo rep *Goodyera repens*, Gym con *Gymnadenia conopsea*, Gym odo *Gymnadenia odoratissima*, Him adr *Himantoglossum adriaticum*, Lim abo *Limodorum abortivum*, Lis cor *Listera cordata*, Lis ova *Listera ovata*, Neo nid *Neottia nidus avis*, Nig bus *Nigritella buschmanniae*, Nig min *Nigritella miniata*, Nig rhe *Nigritella rhellicani*, Oph ber *Ophrys bertolonii*, Oph hol *Ophrys holosericea*, Oph ins *Ophrys insectifera*, Oph sph *Ophrys sphegodes*, Orc mas *Orchis mascula*, Orc mil *Orchis militaris*, Orc mor *Orchis morio*, Orc pur *Orchis purpurea*, Orc sim *Orchis simia*, Orc tri *Orchis tridentata*, Orc ust *Orchis ustulata*, Pla bif *Platanthera bifolia*, Pla chl *Platanthera chlorantha*, Pse alb *Pseudorchis albida*, Tra glo *Traunsteinera globosa*.

Given the large interspecific variation observed within each habitat preference group, we also considered the shift rate along the whole elevational distribution for each species, separately. We assessed how and where orchid elevational distributions shifted and whether this shift led to an overall contraction comparing deciles of the two elevation density distributions^40^. We generally observed asymmetric and idiosyncratic range shifts across species, with only a few species showing a symmetric march upwards of both rear and leading edge (e.g. *Orchis mascula*, *Dactylorhiza sambucina*, *Nigritella rhellicani*). More than 50% of the species were not able to fully track climate change (Fig. 7). Although most forest species did not change their distribution between the two periods, some species (e.g. *Goodyera repens*, *Neottia nidus avis*, *Epipactis muelleri*) showed a downward shift at the leading margin resulting in a range contraction. Only two forest species moved upwards (*Corallorhiza trifida* and *Listera cordata*) but with a slower shift at the leading edge. By contrast, generalists were the only group of orchids that often expanded their range to higher elevations by moving the leading edge faster than the rear edge (e.g. *Cypripedium calceolus*, *Listera ovata*, *Orchis mascula*, *Platanthera bifolia*). All grassland orchids moved significantly upwards; however, three moved quicker at the rear than at the leading edge, therefore contracting their range (*Coeloglossum viride*, *Gymnadenia odoratissima*, *Traunsteinera globosa*). Semi-natural orchids showed either a stable range (e.g. *Himantoglossum adriaticum*) or a downward shift of the leading edge (e.g. *Orchis morio* and *Orchis tridentata*), contracting their range. Except for *Nigritella miniata*, subalpine orchids moved upwards with a trend for a slower leading edge shift (e.g. *Pseudorchis albida*). Finally, two of the four species of wetland orchids shifted significantly upwards. It is important to stress that rare species with low numbers of records were overrepresented in the wetland and semi-natural group affecting the power of the decile comparison described above^40^ (78% of the species with <200 records belonged to wetland and semi-natural group, Supplementary Table 1).

Forest orchids often did not exhibit any shift in their elevational distribution probably because the slow upward movement of forests hindered the range expansion of orchids. Similarly, Lenoir et al.^41^ reported in the French Alps a non-significant upward shift for four species of forest orchids also included in our analysis. Moreover, previous studies indicate that forest ecosystems may buffer the effects of climate change on plants^42–44^, promoting species’ persistence and resulting in a delayed response of plant communities^45^. Species that can colonize grasslands from sea level to the highest elevations shifted their rear edge and optimum faster than most other groups and also faster than regional climate warming. These species possess the widest thermal niche that can help them to rapidly take advantage of warming temperature. Several generalists were able to track climate change probably due to their ability to colonize different habitat types over the entire elevational range. Orchids inhabiting semi-natural grasslands below the tree-line shifted their distribution in the direction opposite to climate change, in fact both the leading edge and the optimum shifted downwards. This shift is consistent with the patterns of land-use changes in the study area where open areas were lost due to the natural downward recolonization by forests^17^, leading to increasingly unfavourable conditions towards the upper limit of the range. Previous studies also found that species may shift downslope as a direct consequence of habitat modification following natural or human-induced disturbances or due to other local changes in habitat suitability^46^. Several cold-adapted orchids of subalpine habitats moved their optima and rear edges upwards quickly, while at the leading edge species failed to colonize novel habitats at the same pace. This is consistent with the slow dynamics of subalpine/alpine habitats related to extreme cold temperature and to geometric constraints of mountain tops, i.e. reduced habitat area^47^. Finally, most orchids occurring in wetlands presented a consistent march upwards of rear and leading edge similar to the speed of temperature warming, possibly indicating that the regional network of protected areas is helping the species to track climate change. In conclusion, in accordance with previous studies evaluating shifts at both rear and leading range limits^22,48^, we observed large interspecific variation that was only partially explained by habitat preference. Idiosyncrasy in range shifts is not consistent with a scenario where temperature is the sole dominant factor driving species range distribution and was previously observed even within species across regions, highlighting how biotic interactions and local, non-thermal abiotic conditions may often supersede the physiological effects of temperature^49^. A full understanding of the response variation among taxa will likely require embracing the complex ways in which species interactions influence range dynamics^39^ and the potential role of microscale variation in climate^43,44^ and habitat quality^50^.

### Study limitations

Several limitations of our study should be kept in mind when interpreting our results. First, resurveying historical plots did not allow us to evaluate colonization dynamics, because we did not sample plots beyond the leading edge of historical species distributions. Second, in the resurvey we only monitored orchids during one growing season in both periods. Although a single visit is generally considered as insufficient to count all species at a site^51^ and among orchid species there is considerable variation in traits that can influence detectability in field observations, our analyses were run at the species level and therefore species detectability should be consistent in the two periods. Third, despite the fine spatial resolution and large sampling effort of our data set, several species were still too rare to robustly evaluate population and range shift dynamics. Fourth, the mechanisms underpinning the observed population decline and range shifts could not be singled out due to the lack of high resolution, historical data on habitat changes beyond the 463 resurveyed sites.

## Conclusions

Except for the most thermophilic species and wetland specialists, we observed population declines, in particular for rear-edge populations. Besides these dramatic demographic trends, different species exhibited idiosyncratic range shifts with >50% of the species not able to fully track climate change. Overall, our results show that only a multi-dimensional approach encompassing local extinction dynamics, local population density and quantification of elevation ranges from rear to leading edges enabled a comprehensive understanding of redistribution dynamics of orchids under global change. At the local scale, in situ management and protection can focus on maintaining habitat quality, while at the regional scale it is crucial to identify and protect habitat patches across elevational ranges to enable species range shifts. Finally, our study highlights the importance of long-term monitoring of rare plant populations and distributions at fine spatial scales^29,35,52,53^, to be able to fully understand and manage the consequences of global change for mountain biodiversity.

## Methods

### Study area

Orchid populations were sampled throughout the Trento Province, NE Italy (6207 km^2^, elevation range 66–3769 m; Fig. 1). The region is located in the centre of the European Alps and represents a hot-spot of plant species diversity, including species whose geographic ranges are Alpine, central and northern European and Mediterranean^54^.

### Climate change

Climate in the region depends primarily on elevation: it is alpine at high elevations and continental in the lowlands. Maximum annual temperature between 1980 and 2010 was 17.5 °C and minimum 7.8 °C (at 200 m a.s.l.)^55^. In Trentino, mean temperatures increased by c. 0.75 °C between 1981 and 2010^55^. A stronger temperature increase was measured during the growing season (spring and summer). A previous study in the region indicated that a vertical spread rate from 3.8 to 5.5 m year^−1^ is necessary for species to be able to fully track climate warming^2^. Precipitation is abundant throughout the year, and mean annual precipitation over the past 40 years was 1050 mm. Annual rainfall slightly increased between 1981 and 2010 (+2%), but decreased in winter (−6%)^55^.

### Land-use

The availability of the major habitat types for orchids is influenced by land-use at different elevations. To describe the current elevational distribution of these major habitat types, we used the most accurate land-use maps available. We used data from the 2009 regional land-use map for alpine habitats, forests and wetlands^56^. For the extent of semi-natural grasslands in 2009, we used a detailed map provided by the Rovereto Museum (provided by F.P. and A.B.). We converted vectorial layers of each habitat into a raster layer with a grain of 50 × 50 m. Then, for each habitat layer we extracted the elevation of each pixel (50 × 50 m) and created a density plot in order to evaluate the regional availability of each habitat type over the elevational gradient (Supplementary Fig. 1). The lowlands were dominated by urban elements and intensively cultivated areas with fragmented semi-natural grasslands (extensively managed or recently abandoned meadows). These habitats historically replaced the native forest vegetation at lower elevations. At mid-elevations, forests interspersed with managed grasslands covered mountain slopes. Above the tree line (1800–2000 m a.s.l.), the landscape was characterized by subalpine grasslands and rocky and snow-covered ground. Wetlands did not exhibit any clear elevational distribution patterns. The study area has experienced two major land-use changes in recent decades. First, forests increased downwards at the expense of open semi-natural areas at mid-elevations (approximately between 600 and 1500 m) due to land abandonment^17^. Currently, forests cover c. 60% of the territory. The abandonment of traditional agriculture is closely linked to demographic changes: human population has decreased >600 m and has increased in the lowlands. Second, agriculture expanded upwards from the lowlands to mid-elevations (up to c. 850 m): the leading edge of grape (c. 750–850 m) and apple cultivation (c. 1000–1100 m) moved upwards in the past two decades^57,58^. These two ongoing changes, of increased direct anthropogenic pressure at low elevations and reduced pressure (abandonment) at mid-elevations, each imposed direct increasing constraints on habitat availability for orchids associated with open areas.

### Historical orchid surveys (1990–2017)

In total, the historical database included 50,074 records belonging to 60 orchid species spanning an elevational gradient from 66 to 2970 m over 28 years (1990–2017) (Fig. 1). However, we present results from analyses of 21,601 sites and 49,303 records for 44 species that meet our criteria for inclusion in the study, i.e. at least 30 records in the first 14 years (1990–2003) and last 14 years (2004–2017) of the historical data set. G.P. and collaborators collected data by sampling the 21,601 sites, systematically covering the whole area of Trento Province. Each site was visited only once. Having identified a potentially suitable area in the field (i.e. natural or semi-natural habitats corresponding to open grassland, wetland or the woodland understorey), using a Global Positioning System (GPS) they marked the site (point), recorded all the orchid species occurring in the close surroundings (c. 50 m) and counted the number of individuals per species. The general small size of orchid populations and the patchy distribution of individuals allowed estimates of population size in the field with relatively low uncertainty. The only exception was when populations were very large. However, the frequency of populations with size >100 individuals was only 4%. The aims of the sampling were to describe the regional orchid species distributions at a very fine spatial resolution and to provide a network of sites to investigate orchids’ population dynamics. The sites were not physically marked as true permanent plots but the centre of each site was georeferenced using a GPS (c. 5–10 m precision) and high-resolution topographical maps. The average density of sample sites was c. 4 per km^2^, including in the count areas where no orchids are usually found (e.g. industrial areas, urban fabric, roads, construction sites, water bodies, cliffs, etc.; Fig. 1). The database is unique in describing the regional distribution of a rare, highly diverse and threatened group of plants because of its massive sampling effort compared to the relatively large spatial and temporal extent, spanning almost three decades. Moreover, the data set covered c. two-thirds of the orchid species occurring across the European Alps^54^. At each site, the following variables were also collected: date of sampling, elevation, detailed site description (vegetation, proximity to roads or constructions, etc.), and slope. Nomenclature follows Perazza and Lorenz^34^. All the data were stored in the private database of G.P. and in the GIS-inventory database of the Museo Civico di Rovereto (Rovereto, Trento, Italy).

### Resurveys (2018–2019)

To detect local extinction of historically recorded populations, we selected a subset of sites to resurvey orchid populations starting from the database described above. The selection of the sites was performed using a stratified random sampling in a GIS environment (QGIS, version 3.6.1-Noosa). The strata were the four major habitat types occurring across the elevational gradient: forests, subalpine areas, semi-natural grasslands, and wetlands. Further criteria of site selection were: (1) to include the whole elevational distribution of each resurveyed species, (2) to exclude sites with the occurrence of a single individual, and (3) to cover most of the geographical area of the historical survey. We revisited 463 sites in all major habitat types, covering the whole elevational range of orchid distributions from the lowlands to high elevation natural areas. Of the final 463 sites, 167 were classified as forests, 53 as subalpine/alpine natural habitats, 198 as semi-natural grasslands and 45 as wetlands. Usually, resurvey studies are constrained by the quality of the baseline data (e.g. relocating the sites), the need to maintain consistent taxonomy and observer effects (e.g. detecting rare species)^59^. In spring and summer 2018 and 2019, G.M. and C.G. revisited the 463 sites following the sampling methodology of the first observer (G.P.), who constantly helped verifying baseline data, confirming species identification, relocating the sites and assessing habitat alterations. The sites were only visited once either in 2018 or in 2019. The resurvey was performed by actively searching the whole area around the sites surveyed in the historical survey (c. 50 m around the originally referenced point). Orchid species and the number of individuals were recorded. Along with the orchid data, the following parameters were recorded: date, elevation, habitat type, and description of any local alteration occurred between the two periods. For the latter, we reported if a local disturbance (e.g. construction sites, touristic activities) or a habitat type change occurred in the second survey by comparing the description of the sites in the initial survey with the current conditions.

### Orchid habitat preference

We attributed each orchid species to one of the six non-overlapping categories using the description of habitat preferences according to Perazza and Lorenz^34^ (*n* = 49 species, Supplementary Table 1). We considered the following categories: (1) specialists of shrubland, broadleaf and conifer forests (forest, *n* = 12 species), (2) generalist species able to colonize both forests and grasslands (generalist, *n* = 9 species), (3) species able to colonize grasslands from low elevations to alpine habitats (grassland, *n* = 6 species), (4) specialists of grasslands below the tree-line including mown meadows, abandoned grasslands, grass margin and extensive perennial crop areas such as vineyards and olive groves (semi-natural grassland, *n* = 5 species), (5) specialists of subalpine open habitats, i.e. rocky habitats, alpine and subalpine grasslands (subalpine, *n* = 5 species), and (6) specialists of wetlands, e.g. fens, mires and ponds (wetland). Due to the well-known habitat specialization of Alpine orchids, there was little uncertainty in the category attribution. To provide an ecological characterization of the habitat categories, we derived for each species Landolt’s indicator values^60^ (Fig. 2) for light, temperature and soil moisture. For each orchid species, we also quantified the realized thermal niche breadth using mean annual temperature (MAT) recorded over 1981–2010 from 21 weather stations in the study area. First, we interpolated the missing temperature values on a layer with 25 m^2^ resolution with the function regression kriging on SAGA using as auxiliary variable elevation obtained from the digital elevation model (EU-DEM Copernicus). Second, we computed the coldest and hottest MAT experienced in the study area as 5 and 95% quantiles of the temperature density distribution. Finally, we calculated thermal niche breadth as the difference between these values. The thermal niche breadths characterize the realized thermal niches for orchid populations in the study area while they are not descriptive of the whole range of temperatures enabling their survival and reproduction.

### Statistical analyses

#### Local survival based on resurveys

We analysed orchid probability of survival across 463 sites, where species were observed in the initial surveys. The response variable was binary assuming the value 1 when the second resurvey reconfirmed the occurrence and 0 when the species was absent. We fitted a generalized linear mixed model (GLMM) with a binomial distribution with species as random factor. We tested as fixed effects time (difference between the year of the initial and second survey, average difference = 20.5 years, SD = 8.4 years), historical population size (number of individuals in the initial survey), habitat alteration (yes or no), the categorical variable of species habitat preference and elevation. Within each species, elevation was standardized to mean 0 and SD = 1 to make the elevational distribution comparable among species and to test whether populations tended to disappear more often at the rear edge than towards the core or upper part of the elevational distribution. This test was valid as the site selection in the resurvey was done to cover the whole elevational distribution of the species included in the analyses. To assess possible collinearity issues between fixed effects, we estimated variance inflation factors (VIFs). VIFs were close to c. 1, indicating very little collinearity among predictors^61^. To match species phenology between the initial and the second survey, we excluded observations with >30-day differences between survey dates. The use of smaller or larger thresholds did not qualitatively change the results. Moreover, we excluded species recorded <5 times in the initial survey (*n* = 43 species) and sites revisited after <5 years. We present results from the full models. We also performed model simplification by removing with a backward deletion procedure non-significant variables (*p* > 0.10). Model estimates between full and reduced models were stable. Recent advances in Bayesian statistics provide efficient methods to model extinction–colonization dynamics^62^. However, these methods rely on the availability of repeated samplings in the same survey period to estimate detectability probabilities. It is important to stress that we had only one visit per period and that our analysis did not focus on estimating real extinction–colonization rates but rather on testing the relative role of different environmental drivers or species traits in explaining population dynamics. Any potential bias in the detectability of the species in the two periods (e.g. different ability between the observers, relocation of the sites) is not expected to be related to any of the tested variables and therefore should not influence the conclusions of our analyses.

#### Temporal trends in population size

To test the effect of time and elevation on orchid population size, we used GLMMs. Within each species, we standardized elevation to mean 0 and SD = 1 to make the elevational distribution comparable among species. We ran separate models for each habitat preference category and considered only species with at least 30 records in the first 14 years (1990–2003) and last 14 years (2004–2017) of the historical data set (*n* = 44 species). We fitted as fixed effects time (continuous), elevation and their interaction using population size as the response variable. Since population size was a count, we used a Poisson distribution. As we expected that population size should be maximum at a thermal or habitat optimum for each species and then decline towards higher and lower elevations, we included the quadratic term of elevation. In all models, we added species as a random intercept, and to correct for overdispersion, we used an observation-level random effect (OLRE) crossed with species^63^. OLRE models the extra Poisson variation in the response variable by using a random intercept with a single level for each data point.

#### Range shifts

Rates of shift in the elevational distribution of species, i.e. changes in optimum, rear (low-elevation) and leading (high-elevation) edge, were computed similarly to Rumpf et al.^13^. To quantify the shift between the recent historical (hereafter ‘historical’) and current range, we split the data set into two periods of 14 years (1990–2003 and 2004–2017). We used time as categorical for two reasons: (1) to minimize the potential bias of botanist sampling effort along the elevation gradient and (2) to obtain solid density distributions to estimate shift of leading and rear edge. Estimating shift at the edge is particularly challenging and therefore pooling 14 years of data allowed to reduce the uncertainty. For each species with >30 records per period, we estimated a density distribution of the elevation of occurrence for the first and second period separately (*n* = 44 species). The rear and leading edge were calculated as the 5 and 95% quantiles of the density distribution and the optimum as the highest peak of the density distribution. The shift was measured by subtracting historical (1990–2003) from current (2004–2017) measures of elevational range. We divided the total shift by 14 years to obtain an annual rate.

To test the effect of habitat preference on the observed shift rates, we fitted three general linear models assuming a Gaussian distribution, testing whether species with different habitat preferences exhibited different mean range shift rates at the rear edge, optimum and leading edge separately. In addition, we carried out post hoc pairwise comparisons using Tukey honestly significant difference with the R package multcomp^64^ to show the differences at rear edge, optimum and leading edge between habitat preference categories. For each species, to further understand where and how the elevational distribution changed in the two periods, we compared the distribution in the historical period with that in the current using the function ‘qcomhd’ of the R package WRS2^65,66^. This function compares deciles estimated from two independent density distributions using a percentile bootstrap to calculate confidence intervals, and therefore, it enables a detailed comparison of shifts along the elevational range. For each species, the analysis can quantify the shifts of the single deciles and if these shifts are different from 0 using bootstrapped intervals of confidence. Low, medium and high deciles approximated rear, optimum and leading edge, respectively. In addition, we tested whether the distribution changed between the historical and the current period using the non-parametric Kolmogorov–Smirnov (K–S) test and adjusting the *p* values with the Benjamini–Hochberg correction. Species showing a significant or marginally significant shift according to K-S test were the same that showed a significant difference between deciles.

#### Potential sampling bias

Since we did not have a fixed network of sites in the two periods, non-random sampling effort across the study region could have biased the estimates of range shift rates^67^. To account for these potential problems, we first described the spatio-temporal patterns of sampling effort. There were roughly the same number of sites sampled in the two periods (10,293 vs. 11,308). We also checked the elevational distribution in each period for all sites and separately for the major habitat types. These analyses did not reveal any strong bias in sampling effort (Supplementary Fig. 2). Our approach of splitting the time series into two periods aimed at comparing two large survey campaigns where sampling was close-to-random in space and time. Second, using the resurvey data from 2018–2019, similarly to Rumpf et al.^13^, we estimated range shift rates for a subset of species (*n* = 20) for which we had at least 10 records in the first and 10 records in the second survey. This approach estimated the rear and leading edge and the optimum using the density distribution based on a spatially fixed network of sites. We calculated shift rates as the difference between current and historical rear edge/optimum/leading edge divided by the average time elapsed between the two surveys within each species (Supplementary Table 4). Then we checked the correlation between range shift rates obtained with the two methods. We found a positive and strong correlation between observed shift rates based on the whole data set and shift rates based on resurveys for the rear shift (*r* = 0.71, *p* < 0.01). For shifts at the leading and optimum, the correlation was still positive but weaker (*r* = 0.38, *p* = 0.10; *r* = 0.39, *p* = 0.09, respectively). Based on the analyses of sampling effort and on the comparison between observed shift rates on the whole data set and shift rates on resurveys, we decided to present the range shifts at rear, leading and optimum positions obtained on the whole data set.

#### Software for statistical analyses

All models were run using GLMMs or GLMs implemented in the package ‘MASS’^68^ and ‘lme4’^69^, while model assumptions were visually evaluated using quantile–quantile plots of the residuals and plots depicting residuals vs. predicted values in the packages ‘DHARMa’ and ‘car’ for R 3.5.1^70^.

### Reporting summary

Further information on research design is available in the Nature Research Reporting Summary linked to this article.

## Supplementary information

Supplementary Information

Reporting Summary

## Data Availability

Data sets as well as R scripts of statistical analyses are published in a publicly available Zenodo digital repository (10.5281/zenodo.4090270).

## Code availability

Data sets as well as R scripts involved in statistical analysis are published in a publicly available Zenodo digital repository (10.5281/zenodo.4090270).
