# Supplementary material for: Consistent population declines but idiosyncratic range shifts in Alpine orchids under global change
Source: Nat Commun. 2020 Nov 17;11:5835. doi: 10.1038/s41467-020-19680-2 (PMC7672077; doi:10.1038/s41467-020-19680-2)
Supplement: Supplementary file 1 — Supplementary Information [file 41467_2020_19680_MOESM1_ESM.pdf]

SUPPLEMENTARY INFORMATION

Consistent population declines but idiosyncratic range shifts in Alpine orchids under global change

Costanza Geppert, Giorgio Perazza, Robert J. Wilson, Alessio Bertolli, Filippo Prosser, Giuseppe Melchiori, Lorenzo Marini

Supplementary Figs. S1–2 and Supplementary Tables S1–4.

SUPPLEMENTARY FIGURES

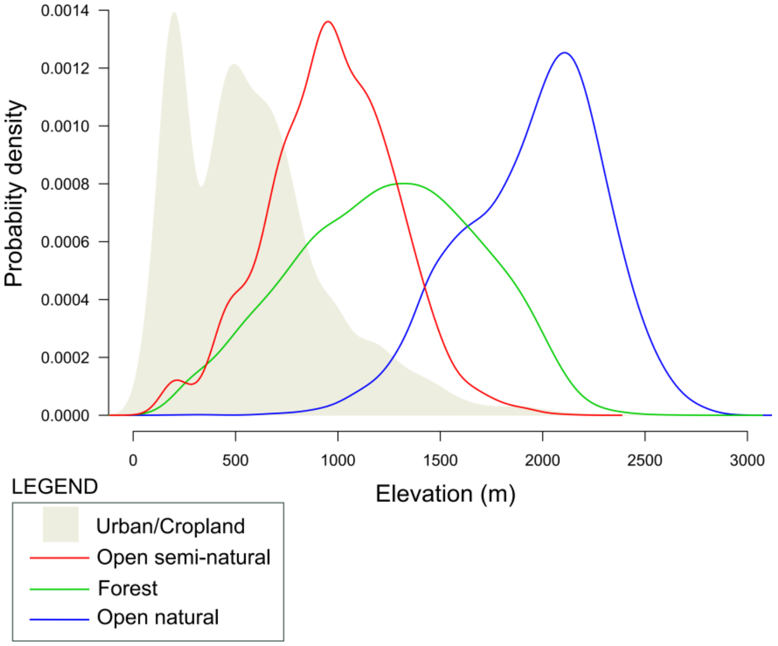

Supplementary Figure 1 Density function of habitat availability at the regional scale over the elevational gradient. Data come from regional land-use maps. Wetlands did not present a clear elevational pattern, i.e. areas were more or less equally distributed across different elevations.

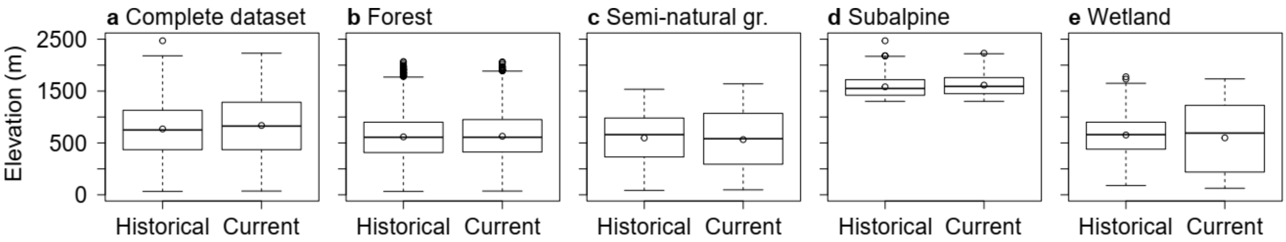

Supplementary Figure 2 Sampling effort over the elevational gradient in both the historical (1990-2003) and current (2004-2017) periods for the complete dataset and for each habitat type. Complete dataset: n = 10,293 historical sites, n = 11,308 current sites. Boxplots display minimum, maximum, median, first quartile, and third quartile and were drawn using the default settings in the boxplot() function in R.

SUPPLEMENTARY TABLES

Supplementary Table 1 List of the orchid species with the number of records in the historical (1990-2003) and current (2004-2017) time periods, habitat preferences, min and max elevation reported in the dataset, and threat category as indicated in the Italian Red List of Threatened Species*. Red List categories as follows, in increasing level of threat: NE – Not Evaluated; LC – Least Concern; NT – Near Threatened; VU – Vulnerable; EN – Endangered. We followed Perazza & Lorenz (2013)[34] for the nomenclature of the species. In the last column, we indicated if the species was included in the population dynamics and range shift analyses (Hist) and/or resurvey analysis (Res).

**Species**	**Count historical**	**Count current**	**Habitat preference**	**Elevation (m)**		**Red list**	**Analysis**
				**Min**	**Max**		
Anacamptis pyramidalis	285	282	Semi-natural	85	1620	NT	Hist, Res
Cephalanthera damasonium	556	547	Forest	140	1650	NE	Hist, Res
Cephalanthera longifolia	1215	1100	Generalist	95	2040	NE	Hist, Res
Cephalanthera rubra	428	228	Forest	125	1700	NE	Hist, Res
Chamorchis alpina	224	163	Subalpine	1580	2660	NE	Hist
Coeloglossum viride	1285	1257	Grassland	570	2970	NE	Hist, Res
Corallorhiza trifida	468	187	Forest	615	2150	NE	Hist, Res
Cypripedium calceolus	556	259	Generalist	380	2295	LC	Hist, Res
Dactylorhiza fuchsii	2840	3759	Generalist	230	2450	NE	Hist, Res
Dactylorhiza incarnata	141	51	Wetland	179	1820	VU	Hist, Res
Dactylorhiza lapponica	94	35	Wetland	400	2145	VU	Hist, Res
Dactylorhiza majalis	66	73	Wetland	1156	2276	NT	Hist, Res
Dactylorhiza sambucina	521	310	Grassland	250	2220	NE	Hist
Epipactis atrorubens	1580	1389	Forest	160	2400	NE	Hist, Res
Epipactis bugacensis	0	82	Wetland	126	212	NE	Res
Epipactis helleborine	1020	1083	Forest	140	1890	NE	Hist, Res
Epipactis muelleri	158	138	Forest	184	1490	NE	Hist, Res
Epipactis palustris	132	48	Wetland	194	1610	NT	Hist, Res
Epipogium aphyllum	120	78	Forest	695	1850	VU	Hist
Goodyera repens	848	359	Forest	250	1960	NE	Hist, Res
Gymnadenia conopsea	2286	1747	Grassland	165	2620	NE	Hist, Res
Gymnadenia odoratissima	827	622	Grassland	100	2500	NE	Hist, Res
Himantoglossum adriaticum	44	203	Semi-natural	270	1030	VU	Hist, Res
Limodorum abortivum	338	267	Forest	66	1180	NE	Hist, Res
Liparis loeselii	16	13	Wetland	437	1018	EN	Res
Listera cordata	383	252	Forest	900	2130	NE	Hist, Res
Listera ovata	1472	950	Generalist	100	2235	NE	Hist, Res
Neottia nidus avis	1392	1440	Forest	140	1820	NE	Hist, Res
Nigritella buschmanniae	51	93	Subalpine	1880	2545	NE	Hist
Nigritella miniata	195	284	Subalpine	1280	2690	NT	Hist
Nigritella rhellicani	1179	1301	Subalpine	1155	2720	NE	Hist, Res
Ophrys apifera	22	70	Semi-natural	98	890	NT	Res
Ophrys bertolonii	70	55	Semi-natural	100	820	EN	Hist, Res
Ophrys holosericea	57	106	Semi-natural	145	1020	VU	Hist, Res
Ophrys insectifera	280	173	Generalist	200	2100	NE	Hist, Res
Ophrys sphegodes	175	134	Semi-natural	85	950	NT	Hist, Res

Orchis coriophora	29	6	Semi-natural	540	1330	NE	Res
Orchis mascula	408	223	Generalist	240	2570	NE	Hist, Res
Orchis militaris	389	174	Semi-natural	100	2140	NT	Hist, Res
Orchis morio	276	188	Semi-natural	90	1470	NT	Hist, Res
Orchis purpurea	121	196	Generalist	140	1500	NT	Hist, Res
Orchis simia	76	63	Semi-natural	90	1040	NT	Hist, Res
Orchis tridentata	429	224	Semi-natural	98	1540	NE	Hist, Res
Orchis ustulata	328	115	Grassland	215	2050	NE	Hist, Res
Platanthera bifolia	1313	1048	Generalist	180	2480	NE	Hist, Res
Platanthera chlorantha	200	67	Generalist	250	1700	NE	Hist, Res
Pseudorchis albida	977	1210	Subalpine	900	2705	NE	Hist, Res
Spiranthes spiralis	14	32	Semi-natural	90	790	NE	Res
Traunsteinera globosa	578	441	Grassland	375	2560	NE	Hist

*IUCN & Società Botanica Italiana. Lista rossa della flora Italiana. 58 (2013).

Supplementary Table 2 Effect of time (difference in years between the two surveys), historical population size, habitat preference, elevation, and habitat alteration (yes and no) on the probability that the orchid population survived until the resurvey. We fitted generalized linear mixed models assuming a binomial distribution with species as random factor (see Methods). Elevation was standardized to mean 0 and SD 1 to make elevational ranges comparable among species.

	**Estimate**	**SE**	**t**	**p**
Time	-0.094	0.086	-1.104	0.269
log (Size)	0.521	0.088	5.932	<0.001
Generalist	0.140	0.254	0.551	0.582
Grassland	-0.758	0.386	-1.962	0.050
Semi-natural grassland	-0.413	0.258	-1.602	0.109
Subalpine	-0.386	0.496	-0.778	0.437
Wetland	-0.148	0.388	-0.380	0.704
Elevation	0.274	0.092	2.984	0.003
Habitat alteration	-0.539	0.174	-3.098	0.002

Supplementary Table 3 Effect of time (year 1990-2017), elevation and their interaction on population size within each habitat preference category. Elevation was standardized to mean 0 and SD 1 to make elevational ranges comparable among species. We fitted generalized linear mixed models assuming a Poisson distribution with species and OLRE as random effects (see Methods). All variables were scaled.

	**Fixed factor**	**Estimate**	**SE**	**t**	**p**
Forest	Elevation^2	-0.123	0.012	-10.546	<0.001
	Elevation	0.028	0.012	2.418	0.016
	Time	-0.163	0.012	-14.074	<0.001
	Elevation x Time	0.036	0.012	3.098	0.002
Generalist	Elevation^2	-0.212	0.013	-16.879	<0.001
	Elevation	-0.093	0.012	-7.479	<0.001
	Time	-0.222	0.013	-17.610	<0.001
	Elevation x Time	0.023	0.013	1.841	0.066
Grassland	Elevation^2	-0.236	0.020	-11.890	<0.001
	Elevation	0.123	0.019	6.403	<0.001
	Time	-0.140	0.019	-7.515	<0.001
	Elevation x Time	0.048	0.019	2.532	0.011
Semi-natural grassland	Elevation^2	-0.095	0.030	-3.129	0.002
	Elevation	-0.172	0.030	-5.763	<0.001
	Time	0.023	0.030	0.749	0.454
	Elevation x Time	0.016	0.031	0.511	0.609
Subalpine	Elevation^2	-0.175	0.026	-6.807	<0.001
	Elevation	0.066	0.025	2.690	0.007
	Time	-0.253	0.025	-10.266	<0.001
	Elevation x Time	0.049	0.024	2.022	0.043
Wetland	Elevation^2	-0.136	0.074	-1.836	0.066
	Elevation	0.106	0.073	1.451	0.147
	Time	0.015	0.074	0.204	0.839
	Elevation x Time	0.086	0.075	1.153	0.249

Supplementary Table 4 Range shifts (m year^{-1}) calculated on resurveyed species with at least 10 records per survey.

**Species**	**Rear edge**	**Optimum**	**Leading edge**	**Count Initial survey**	**Count Second survey**
Anacamptis pyramidalis	2.307	3.402	-4.566	52	38
Cephalanthera damasonium	1.19	1.826	-4.583	28	30
Cephalanthera longifolia	-0.758	10.343	-9.273	74	46
Cephalanthera rubra	8.284	6.285	2.334	24	18
Coeloglossum viride	25.528	29.605	4.376	27	12
Dactylorhiza fuchsii	2.958	-1.234	-3.614	96	76
Dactylorhiza incarnata	2.915	2.334	8.177	40	19
Epipactis atrorubens	3.403	3.666	6.354	56	36
Epipactis helleborine	-0.597	26.613	-4.22	56	45
Gymnadenia conopsea	15.101	42.903	-2.055	80	45
Himantoglossum adriaticum	-7.841	-4.428	1.398	26	13
Limodorum abortivum	1.093	0.201	-13.158	37	26
Listera ovata	-1.932	0.529	8.231	50	43
Neottia nidus avis	6.049	23.805	0.643	60	38
Nigritella rhellicani	2.878	2.748	7.459	25	16
Orchis morio	-2.582	-3.048	-7.309	54	22
Orchis purpurea	9.324	7.405	-8.974	14	10
Orchis tridentata	-2.068	12.285	-1.235	47	25
Platanthera bifolia	5.761	1.688	-8.362	64	24
Pseudorchis albida	14.562	-1.699	2.381	29	14
