# Supplementary material for: Consistent population declines but idiosyncratic range shifts in Alpine orchids under global change
Source: Nat Commun. 2020 Nov 17;11:5835. doi: 10.1038/s41467-020-19680-2 (PMC7672077; doi:10.1038/s41467-020-19680-2)
Supplement: Supplementary file 2 — Reporting Summary [file 41467_2020_19680_MOESM2_ESM.pdf]

nature research

Corresponding author(s): Costanza Geppert, Lorenzo Marini

Last updated by author(s): Oct 12, 2020

# Reporting Summary

Nature Research wishes to improve the reproducibility of the work that we publish. This form provides structure for consistency and transparency in reporting. For further information on Nature Research policies, see our Editorial Policies and the Editorial Policy Checklist.

## Statistics

For all statistical analyses, confirm that the following items are present in the figure legend, table legend, main text, or Methods section.

| n/a | Confirmed | |
|---|---|---|
| ☐ | ☒ | The exact sample size (*n*) for each experimental group/condition, given as a discrete number and unit of measurement |
| ☐ | ☒ | A statement on whether measurements were taken from distinct samples or whether the same sample was measured repeatedly |
| ☐ | ☒ | The statistical test(s) used AND whether they are one- or two-sided<br>*Only common tests should be described solely by name; describe more complex techniques in the Methods section.* |
| ☐ | ☒ | A description of all covariates tested |
| ☐ | ☒ | A description of any assumptions or corrections, such as tests of normality and adjustment for multiple comparisons |
| ☐ | ☒ | A full description of the statistical parameters including central tendency (e.g. means) or other basic estimates (e.g. regression coefficient) AND variation (e.g. standard deviation) or associated estimates of uncertainty (e.g. confidence intervals) |
| ☐ | ☒ | For null hypothesis testing, the test statistic (e.g. *F*, *t*, *r*) with confidence intervals, effect sizes, degrees of freedom and *P* value noted<br>*Give P values as exact values whenever suitable.* |
| ☒ | ☐ | For Bayesian analysis, information on the choice of priors and Markov chain Monte Carlo settings |
| ☐ | ☒ | For hierarchical and complex designs, identification of the appropriate level for tests and full reporting of outcomes |
| ☒ | ☐ | Estimates of effect sizes (e.g. Cohen's *d*, Pearson's *r*), indicating how they were calculated |

*Our web collection on statistics for biologists contains articles on many of the points above.*

## Software and code

Policy information about availability of computer code

| | |
|---|---|
| Data collection | Re-survey coordinates were determined using QGIS, version 3.6.1-Noosa |
| Data analysis | All analyses were conducted in the programming environment R version 3.5.1. The following packages were used: "MASS","lme4" , "car", "DHARMa", "multcomp"and "WRS2". To produce plots the following packages were used: "ggplot2", "visreg" and "effects". See Methods for further details. |

For manuscripts utilizing custom algorithms or software that are central to the research but not yet described in published literature, software must be made available to editors and reviewers. We strongly encourage code deposition in a community repository (e.g. GitHub). See the Nature Research guidelines for submitting code & software for further information.

## Data

Policy information about availability of data

All manuscripts must include a data availability statement. This statement should provide the following information, where applicable:

- Accession codes, unique identifiers, or web links for publicly available datasets
- A list of figures that have associated raw data
- A description of any restrictions on data availability

Datasets, as well as R scripts of statistical analyses are published in a publicly available Zenodo digital repository (http://doi.org/10.5281/zenodo.4090270).

# Field-specific reporting

Please select the one below that is the best fit for your research. If you are not sure, read the appropriate sections before making your selection.

☐ Life sciences ☐ Behavioural & social sciences ☒ Ecological, evolutionary & environmental sciences

For a reference copy of the document with all sections, see nature.com/documents/nr-reporting-summary-flat.pdf

# Ecological, evolutionary & environmental sciences study design

All studies must disclose on these points even when the disclosure is negative.

| | |
|---|---|
| Study description | We analysed population survival, trends in population size, and range shift dynamics of Alpine orchids over 28 years in one of the plant diversity hot-spots of Europe. The historical dataset included 50,074 records for 60 orchid species recorded across the entire orchid elevational range of an Alpine region (66-2,970 m) over 28 years. However, we present results from analyses with 49,303 records for 44 species that meet our criteria for inclusion in the study. In addition, we selected a subset of sites (463) to resurvey orchid populations to detect evidence of survival or local extinction. |
| Research sample | The historical dataset on orchid occurrence and abundance was collected by GP (Giorgio Perazza) and collaborators. All the data were stored in the private database of GP and in the GIS-inventory database of the Museo Civico di Rovereto (Rovereto, Trento, Italy). Orchids are one of the most threatened groups of plants and population declines are well documented worldwide. However, previous research on mountain floras has focused on warming impacts on common taxa, while the response of rare and threatened species to global change has been largely overlooked due to data limitations. Our database is unique in describing the regional distribution of a rare, highly diverse and threatened group of plants because of its massive sampling effort compared to the relatively large spatial and temporal extent, spanning almost three decades. |
| Sampling strategy | The aims of the sampling of the historical database were to describe the regional orchid species distribution at a very fine spatial resolution, and to provide a network of sites to investigate orchids' population dynamics. In 2018, starting from the historical database, we selected a subset of sites to resurvey orchid populations to detect evidence of survival or local extinction. The selection of the sites was performed using a stratified random sampling in a GIS environment. The strata were the four major habitat types occurring across the elevational gradient: forests, alpine areas, semi-natural grasslands and wetlands. Further criteria of site selection were: 1) to include the whole elevational distribution of each resurveyed species, 2) to exclude sites with the occurrence of a single individual, 3) to cover most of the geographical area of the historical survey. |
| Data collection | GP and collaborators collected data by sampling 21,601 sites, systematically covering the whole area of Trento Province. Each site was visited only once. Having identified a potentially suitable area in the field (i.e. natural or semi-natural habitats corresponding to open grassland, wetland or the woodland understorey), using a GPS they marked the site (point), recorded all the orchid species occurring in the close surroundings (c. 50 m), and counted the number of individuals per species. The general small size of orchid populations and the patchy distribution of individuals allowed estimates of population size in the field with relatively low uncertainty. The sites were not physically marked as true permanent plots but the centre of each site was georeferenced using a GPS (c. 5-10 m precision) and high-resolution topographical maps. At each site, the following variables were also collected: date of sampling, elevation, detailed site description (vegetation, proximity to roads or constructions etc.) and slope. All the data were stored in the private database of GP and in the GIS-inventory database of the Museo Civico di Rovereto (Rovereto, Trento, Italy). In spring and summer 2018 and 2019, GM and CG revisited the 463 sites following the sampling methodology of the first observer (GP), who constantly helped verifying baseline data, confirming species identification, relocating the sites and assessing habitat alterations. The sites were only visited once either in 2018 or in 2019. The re-survey was performed by actively searching the whole area around the sites surveyed in the historical survey (c. 50 m around the originally referenced point). Orchid species and number of individuals were recorded. Along with the orchid data, the following parameters were recorded: date, elevation, habitat type, and description of any local alteration occurred between the two periods. For the latter, we reported if a local disturbance (e.g. construction sites, touristic activities) or a habitat type change occurred in the second survey by comparing the description of the sites in the initial survey with the current conditions. |
| Timing and spatial scale | The historical dataset was collected starting from 1990 to 2017, while the re-survey took place in spring and summer 2018-2019. Data collection took place on a daily basis as long as weather conditions permitted it. Orchid populations were recorded throughout Trento Province, NE Italy (6207 km2, elevation range 66-3769 m), the historical dataset comprehends 21,601 sites, while te re-survey 463 sites. |
| Data exclusions | 1) To reduce model uncertainty, we excluded species with less than 30 records in both historical and current periods when testing the effects of elevation and time on population size and when computing range shift; 2) From re-survey analyses, a) we excluded observations with more than 30 day differences between surveys in the two periods to match species phenology, b) we excluded 11 observations for 9 species recorded less than 5 times (either singletons or doubletons) in the initial surveys because models included species as random factor and we could not test our covariates at the species level, and c) we excluded sites revisited after less than 5 years. The original design of the resurveys aimed at comparing historical vs. current conditions using this threshold. However, during the re-surveys some sites were resurveyed just because they were close to others matching our inclusion criteria. Therefore, they were excluded for consistency. However, results would have not changed by including these sites; 3) to estimate range shift rates with relatively low uncertainty using the network of re-surveyed sites, we excluded species with less than 10records in the first and 10 records in the second survey. |
| Reproducibility | Not applicable since our study is not experimental (i.e. manipulative). |
| Randomization | 1) Randomization is not relevant for our study when we analysed the historical database. However, we performed in-depth analysis |

| | |
|---|---|
| | of potential sampling biases in the botanists' surveys; 2) In the resurveys, we randomized site selection within the strata (i.e. habitat types). |
| Blinding | Blinding is not relevant for our study as we analysed an existing database. |

Did the study involve field work? ☒ Yes ☐ No

## Field work, collection and transport

| | |
|---|---|
| Field conditions | Fieldwork was carried out from 1990 to 2019. Climatic conditions such as temperature and rainfall varied on a day to day basis. In the study area, maximum annual temperature between 1980 and 2010 was 17.5 °C and minimum 7.8 °C (at 200 m a.s.l.). Precipitation is abundant throughout the year, and mean annual precipitation over the last 40 years was 1050 mm. |
| Location | The study was conducted in the Province of Trento, NE Italy (6207 km2, elevation range 66-3769 m). The region is located in the North-East part of the European Alps, and represents a hot-spot of plant species diversity. |
| Access & import/export | During the fieldwork, we actively searched the whole area around the sampling sites. We did not collect any sample instead we recorded orchid species and number of individuals together with environmental variables. |
| Disturbance | No disturbance was caused by the study. |

# Reporting for specific materials, systems and methods

We require information from authors about some types of materials, experimental systems and methods used in many studies. Here, indicate whether each material, system or method listed is relevant to your study. If you are not sure if a list item applies to your research, read the appropriate section before selecting a response.

### Materials & experimental systems

| n/a | Involved in the study | |
|---|---|---|
| ☒ | ☐ | Antibodies |
| ☒ | ☐ | Eukaryotic cell lines |
| ☒ | ☐ | Palaeontology and archaeology |
| ☒ | ☐ | Animals and other organisms |
| ☒ | ☐ | Human research participants |
| ☒ | ☐ | Clinical data |
| ☒ | ☐ | Dual use research of concern |

### Methods

| n/a | Involved in the study | |
|---|---|---|
| ☒ | ☐ | ChIP-seq |
| ☒ | ☐ | Flow cytometry |
| ☒ | ☐ | MRI-based neuroimaging |
